# A Treatment Approach for Carotid Blowout Syndrome and Soft Tissue Reconstruction after Radiotherapy in Patients with Oral Cancer: A Report of 2 Cases

**DOI:** 10.3390/jcm12093221

**Published:** 2023-04-30

**Authors:** Tobias Moest, Marco Rainer Kesting, Maximilian Rohde, Werner Lang, Alexander Meyer, Manuel Weber, Rainer Lutz

**Affiliations:** 1 Department of Oral and Maxillofacial Surgery, University Hospital Erlangen, Glückstraße 11, 91054 Erlangen, Germany; 2 Department of Vascular Surgery, University Hospital Erlangen, Krankenhausstraße 12, 91054 Erlangen, Germany

**Keywords:** carotid blow out syndrome, vessel graft, vessel-depleted neck, irradiation

## Abstract

Background: This retrospective case series study aims to demonstrate a salvage technique for the treatment of carotid blow-out syndrome (CBS) in irradiated head and neck cancer patients with a vessel-depleted neck. Methods: Between October 2017 and October 2021, two patients (N = 2) with CBS were treated at our institution in a multidisciplinary approach together with the Department of Vascular Surgery. Patients were characterized based on diagnoses, treatment procedures, and the subsequent postoperative course. Results: Surgical emergency intervention was performed in both cases. The transition zone from the common carotid artery (CCA) to the internal carotid artery (ICA) was resected and reconstructed with a xenogic (case 1) or autogenic (case 2) interposition (end-to-end anastomosis). To allow reconstruction of the vascular defect, an additional autologous vein graft was anastomosed to the interposition graft in an end-to-side technique, allowing arterial anastomosis for a free microvascular flap without re-clamping of the ICA. Because of the intraoperative ICA reconstruction, none of the patients suffered a neurological deficit. Conclusions: The techniques presented in the form of two case reports allow for acute bleeding control, cerebral perfusion, and the creation of a vascular anastomosis option in the vessel-depleted neck.

## 1. Introduction

Carotid blowout syndrome (CBS) is a rare and life-threatening complication in patients treated for head and neck cancer, most commonly with a history of prior surgery and radiotherapy. CBS is the result of vessel wall necrosis that can occur as a result of surgical and adjuvant tumor therapies, chronic inflammation, and fistula.

The general incidence of CBS in oncological procedures in the head and neck region is 3–4.5% [1,2], while the incidence in patients following previous radiotherapy varies from 4.5% to 21.1% [3,4]. According to Macdonald et al., the risk of CBS increases by a factor of 7.6 in patients with head and neck tumors [5].

Carotid artery rupture occurs mainly in the common carotid artery (CCA), near the bifurcation (60–70% of cases). A much smaller proportion also occurs in the internal carotid artery (ICA) [6,7,8]. In general, CBS is more common in atherosclerotic vessel segments with stenosis [9].

The main risk factor for the development of CBS is previous radiation therapy after tumor surgery [10]. Patients who have received more than 70 Gy have an up to 14-fold increased risk of developing CBS [11]. From a histopathological point of view, radiotherapy leads to the formation of free radicals, which in turn favor thrombosis, obliteration of the vasa vasorum of the vascular adventitia, and vascular fibrosis. The result is premature atherosclerotic change and significant vascular weakness. CBS is therefore the result of vascular weakness due to ischemia resulting from adventitial insufficiency [10].

Based on this, it is understandable that surgical procedures that result in damage to the adventitia increase the likelihood of CBS. Patients who underwent neck dissection surgery showed an eight-fold increased risk of CBS [11]. In particular, patients with recurrent tumors after previous surgical treatment with neck dissection and adjuvant radiotherapy, where additional radiotherapy is required if reoperation is not possible, are at particularly high risk of developing CBS.

The general incidence of CBS in re-irradiated patients with tumor recurrence is 0–17%. In these cases, tumor ingrowth into the CCA is an additional risk factor for CBS [12,13]. In general, the median cumulative dose of both radiotherapy modalities is between 110 and 130 Gy. Studies show an increased rate of CBS when the cumulative dose is >130 Gy [10].

Another risk factor is chronic inflammation in the sense of bacterial infection, which leads to thrombosis of the vasa vasorum of the arterial wall and has an increased susceptibility to the negative influence of inflammatory mediators in contaminated wound areas [6]. In addition to post-operative wound infections, oro- or pharyngocutaneous fistulae pose a high risk due to tryptic enzyme activity. Permanent contact with saliva leads to digestion of the arterial wall by tryptic enzymes, and bleeding may be provoked. Powitzky et al. showed in their study that 38% of CBS cases demonstrated inflammation, 40% fistula, and 55% tissue necrosis [6].

Due to the extremely high mortality rate of 76%, CBS is a feared complication [14]. In emergencies, CBS is treated by ligation of the CCA or ICA without consideration of collateral cerebral circulation, increasing the risk of neurological morbidity [15]. In the literature, mortality rates vary from 15–100%, with an average of 50% [10]. In addition to surgical treatment of CBS, endovascular interventions, including vascular plugging and covered stent repair, are considered feasible therapeutic options [11,16].

The aim of the present study was to present clinical examples of techniques that can be used for the surgical management of spontaneous and intraoperative CBS. The techniques presented in the form of two case reports allow acute hemorrhage control during permanent cerebral perfusion (intraluminal shunt) and the creation of a vascular anastomosis option in the avascular neck.

## 2. Patients, Materials and Methods

This interdisciplinary retrospective case series was performed at the Department of Oral and Maxillofacial Surgery in cooperation with the Department of Vascular Surgery (University Clinic Erlangen). Patients were identified by screening the digital clinic documentation system (MCC^®^, Meierhofer AG, Munich, Germany) and the digital patient files (Soarian Clinicals^®^, Cerner Health Services, Erlangen, Germany; Meona^®^, Meona GmbH, Freiburg, Germany).

Two patients with CBS were included in this retrospective analysis between October 2017 and October 2021. The Ethics Committee of the University Hospital Erlangen was consulted regarding the approval of the study. The committee decided that ethics approval was not required for retrospective case analyses.

## 3. Results

### 3.1. Case 1 (Spontaneous CBS)

In July 2017, a 76-year-old female Caucasian patient presented to our clinic with a recurrence of oral squamous cell carcinoma (OSCC) with infiltration of the mandible. She had previously undergone resection of an OSCC on the left cheek (2008, pT1 pN0 M0 G2 L0 V0 R0) and a second OSCC in the alveolar bone of the left mandible (2014, pT1 pN1 (1/1, perinodal) L0 V0 Pn0 G2 R0). Postoperative interstitial brachytherapy with up to 50 Gy in 2008/2009 and definitive concurrent radiotherapy (2014, total dose 64 Gy) and chemotherapy with 5-FU and cisplatin (10–12/2014) with maintenance chemotherapy with cisplatin (01/2015) were initially performed. In 10/2015, a recurrent tumor of the OSCC in the area of the anterior floor of the mouth and the alveolar crest of the left lower jaw (rpT4a pN0 (0/15) L0 V0 Pn0 G2 infiltration depth 0.5 cm, R0) was resected and reconstructed with a microvascular anastomosed upper arm graft.

The present recurrent tumor (rpT4a L0 V0 Pn0 G3 Rx), newly detected in July 2017, was treated with a partial mandibular resection (continuity resection). The mandible was reconstructed with a reconstruction plate (Stryker GmbH & Co. KG, Duisburg, Germany, 2.7 mm) and a free microvascular M. latissimus dorsi flap. The postoperative course was very complicated due to recurrent circulatory disturbances of the flap and cervical wound healing disorders, which were covered by a musculocutaneous anteriolateral thigh flap (ALT).

During the postoperative inpatient period, spontaneous erosive bleeding of the left CCA/ICA required emergency surgery. After systemic heparinization and clamping, the ruptured carotid bifurcation was resected, and the ICA was reconstructed with an on-table pericardial tube graft (bovine pericardium; PeriGuard Repair Patch^®^ (Synovis, St. Paul, MN, USA), as autologous vessel grafts of adequate size were not available.

**Figure 1:**
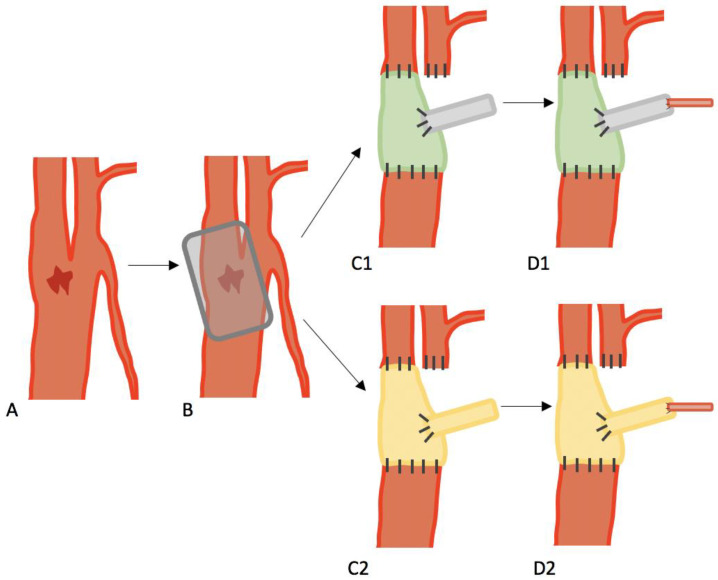
(**A**) Shows the typical location of carotid blow-out syndrome, which is often located at the junction of the CCA and ICA. (**B**) Shows the resected portions of the CCA, ICA, and ECA. (**C1**,**C2**) Illustrates the different reconstruction techniques: (**C1**) with a xenogenic graft (green) combined with an autologous second graft (grey); (**C2**) with an autologous graft (yellow) combined with an autologous second graft (yellow); (**D1**,**D2**) shows the anastomosis of the autologous graft (grey in (**D1**) and yellow in (**D2**)) with the arterial branch of the graft.

**Figure 2:**
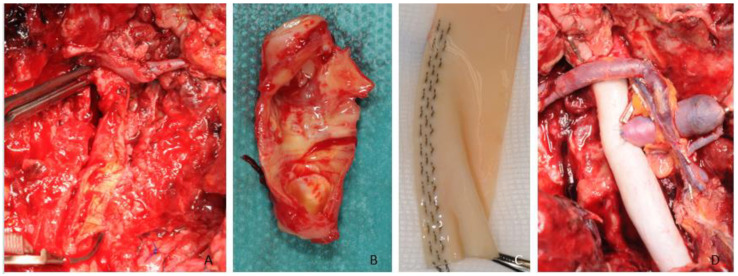
(**A**) Shows the intraoperative situation of the ruptured CCA near the bifurcation. (**B**) Resection of the CCA and the bifurcation to the ECA/ICA. (**C**) Individually table-made xenogenic interponate. (**D**) Anastomosed graft to the ICA/CCA with an additional graft for the microvascular free flap artery.

The tube graft was constructed with a linear stapler and anastomosed to the CCA and ICA by end-to-end anastomosis. As necrotic parts of the previous flap had to be removed, an approximately 3 cm-long arm vein graft (the cephalic vein of the right upper arm) was placed end-to-side on the pericardium to allow arterial anastomosis of a soft tissue graft. An upper arm flap was used to fill the cervical soft tissue defect resulting from the resection of the necrotic tissue mentioned above (Figure 1(D1) and Figure 2).

The patient was discharged from the hospital on the 36th day after the operation. After discharge, the patient attended our six-weekly tumor follow-up in our outpatient clinic.

### 3.2. Case 2 (Intraoperative CBS)

A 65-year-old Caucasian male presented to our clinic in February 2021 with a suspected malignancy at the floor of the mouth, a significantly worsened general condition, and a weight loss of 10 kg within a few weeks. The staging CT scan showed a cT4a N2b OSCC on the right floor of the mouth. In March 2021, surgery was performed with a temporary tracheotomy, neck dissection of levels I-III on both sides, and resection of the floor of the mouth and the mandible from the left to right jaw angle. The mandible was reconstructed with a reconstruction plate (Stryker GmbH & Co. KG, Duisburg, Germany, 2.7 mm) in combination with a microvascular latissimus dorsi transplant, as osseous reconstruction was rejected by the patient.

Histopathological evaluation revealed a pT4a pN2c (2/43) L0 V0 Pn1 OSCC with bone infiltration of 1.4 cm, G2, R0. Due to the tumor size and lymph node involvement, the postoperative interdisciplinary tumor board recommended adjuvant radiochemotherapy to optimize tumor control. However, the planning CT showed signs of early recurrence, and definitive radiotherapy (increased final dose) and adjuvant chemotherapy were recommended, which were delivered from May 2021 to July 2021 with a total cumulative dose of 70 Gy to the oral floor and lymphatic drainage target volume. The recommended concurrent chemotherapy was rejected by the patient.

However, during the course of radiotherapy, a significant intraoral dehiscence with significant exposure of the reconstruction plate was observed, leading to severe aesthetic and functional impairment. Due to the risk of further complications, removal of the reconstruction plate was recommended. The patient has now requested osseous reconstruction of the mandible for future implant-prosthetic rehabilitation. Removal of the reconstruction plate alone, with the risk of permanent tracheostomy due to possible soft tissue collapse, was categorically rejected.

Because of the expected intraoperative soft tissue deficit due to previous radiotherapy, extraoral cervical soft tissue reconstruction with a microvascular ALT transplant in combination with a free fibula transplant was planned.

The procedure was performed using a “three-team approach”. One team prepared the cervical vessels and removed the osteosynthesis material, while the second and third teams harvested the ALT and the free fibula transplant.

The preparation of the neck was extremely difficult due to extreme fibrosis of the soft tissues and significant scarring. Despite careful preparation, a wall weakness of the CCA was observed, leading to a wall defect with massive bleeding (CBS). As direct suture reconstruction was not possible, a vascular surgeon was consulted at once.

**Figure 3:**
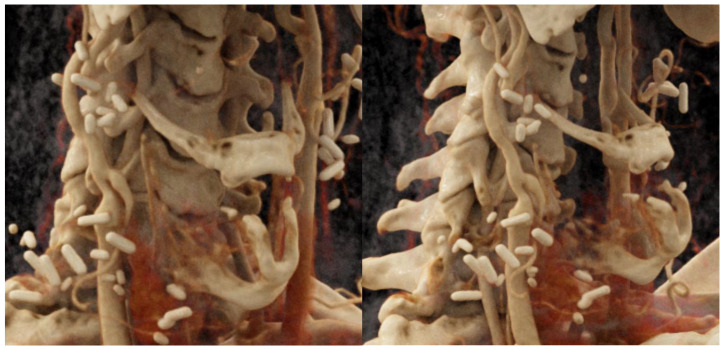
Three-dimensional reconstruction of the post-operative situation by angiography showing the interponate connecting the CCA to the ICA and the transition from the additional interponate to the arterial branch to the graft in case 2.

Systemic heparinization was initiated, followed by preparation of the carotid bifurcation and ligation of the external carotid artery (ECA). The area of perforation, which included parts of the ICA and parts of the CCA, was resected. To ensure cerebral perfusion, an intraluminal shunt system (FlexcelTM Carotid Shunt; Version 2020-05; LeMaitre Vascular; Burlington, MA, USA) was placed until an approximately 5 cm long venous segment of the greater saphenous vein was prepared and anastomosed to the ICA and CCA as an arterial interposition (end-to-end). To allow microvascular reconstruction of the cervical soft tissue dehiscence, an additional 2 cm-long vein graft was anastomosed to the interponate in an end-to-side technique, which could be used for arterial anastomosis of the ALT graft. This avoided further cross-clamping of the ICA during microsurgery. Due to the desolate cervical vascular status, the fibular graft was not completely harvested, transferred back to the lower leg, and replanted with osteosynthesis. The postoperative course was uneventful. No neurological deficit was noted. The patient was discharged on postoperative day 13 with a permanent tracheostomy tube. Postoperative angiography of the cervical vessels showed adequate perfusion (Figure 3). Postoperative care was provided in our outpatient clinic. After discharge, the patient attended our six-week tumor follow-up in our outpatient clinic.

## 4. Discussion

The aim of this case series was to demonstrate techniques that allow for the management of CBS while at the same time enabling microvascular soft tissue reconstruction of cervical defects using free flaps. Two patient cases are presented.

The two cases have in common that the preoperative anatomical and soft tissue situations were extremely challenging. Both cases showed previous operations, wound infection/dehiscence, a previously irradiated neck (cases 1 + 2) and partial microvascular free flap necrosis (case 1). Both cases presented with CBS and cervical soft tissue defects too large for local management while at the same time presenting “vessel depleted necks” with no suitable vessels for microvascular anastomosis.

The literature describes different techniques for the handling of mass bleeding resulting from the erosion of the carotid bifurcation. The number of treatment techniques for acute CBS is more limited. Here, vascular ligation represents the ultima ratio, and a series of possible consequences should be considered. The direct neurologic consequences of ligation of the ICA are not predictable, and range from an asymptomatic course to a disabling major stroke [17].

Patients with a high risk for CBS often also show a “vessel depleted neck” due to multiple prior surgical and radiochemical interventions. In the same patient group, as is the case here, a microcvascular flap treatment may be necessary due to the size of the defect. In order to make microvascular tissue transfer possible, individual solutions range from simple interposition grafts to extracorporeal perfusion devices [18,19,20,21,22,23,24].

As the standard vascular network defined by the ECA branches and the jugular vein is inaccessible in those cases, other recipient vessels for microvascular anastomoses have to be chosen.

For arterial anastomoses, branches of the subclavian (internal mammary artery and thyrocervical trunk) and axillary arteries (thoracoacromial artery) as well as the superficial temporal artery have been described in the literature [25,26,27].

For venous drainage, the accessory veins of the target artery can be prepared. Another option is to use the cephalic vein because of its reliable drainage, consistent anatomy, long pedicle, and high flow to the low-pressure system [18].

As mentioned above, CBS can occur under unfavorable conditions that require new strategies.

In addition to patient survival, the aim is to solve the acute bleeding problem, provide an anastomosis option for microsurgical defect treatment, and allow sufficient cerebral perfusion to prevent neurological damage. All these requirements were met with the two techniques described in this report.

In both cases, the choice of vascular graft depended on the presence/location of a suitable donor site, e.g., the presence of a microvascular donor site despite the “vessel depleted neck”.

In case 1, a xenograft was used to manufacture a neo-ICA. In case 2, V. saphena magna was harvested by extending the surgical access to the ALT flap. The xenograft could be manufactured in length and diameter as required. The saphenous vein has nearly the same caliber as the ICA, and the length could be varied as required. This way, graft positioning was unproblematic, and no risk of kinking existed.

In case 2, a collateral circulation bridging the resected CCA/ICA gap was created using an intraluminal shunt. Installing the shunt takes about 5–8 min when performed by an experienced surgeon. During this period, the CCA as well as the ICA/ECA are clamped. The anastomosis of the additional lateral graft to the interposition graft for subsequent arterial flap anastomosis was performed before re-opening the clamps in order to avoid additional clamping of the interposition graft.

With the techniques described, simultaneous management of both acute bleeding (CBS) and soft tissue reconstruction was possible. In the treatment of the CBS, an almost optimal situation for microsurgical transplant anastomosis was constructed. Both patients experienced no further bleeding or neurological complications; cervical soft tissue reconstruction was successful.

## 5. Conclusions

ICA/CCA reconstruction with an autologous or xenogeneic vascular graft and an additional vascular branch for subsequent microvascular flap anastomosis is an effective method for the combined treatment of CBS and cervical soft tissue defects in patients with a “vessel depleted neck”.

## Data Availability

Additional chart data from all patients may be provided by contacting the corresponding author.
